# No Established Link between Repeated Transient Chokes and Chronic Traumatic Encephalopathy Related Effects. Comment on Lim, L.J.H. et al. Dangers of Mixed Martial Arts in the Development of Chronic Traumatic Encephalopathy. *Int. J. Environ. Res. Public Health* 2019, *16*, 254

**DOI:** 10.3390/ijerph16061059

**Published:** 2019-03-23

**Authors:** Samuel J. Stellpflug

**Affiliations:** Regions Hospital Department of Emergency Medicine, 640 Jackson St., Saint Paul, MN 55101, USA; samuel.j.stellpflug@healthpartners.com

**Keywords:** choke, strangle, asphyxia, rear naked, jiu jitsu, MMA

## Abstract

This letter to the editor is in response to “Dangers of Mixed Martial Arts in the Development of Chronic Traumatic Encephalopathy” by authors Lim, Ho, and Ho, which was published in the International Journal of Environmental Research and Public Health (2019; 16: 254). This communication clarifies some potentially misleading word choices by the authors and addresses the insinuated, but not established, link between repeated transient choking episodes during martial arts training and a gradual decline in neuropsychiatric testing in the patient presented in the report.

I have reviewed the article “Dangers of Mixed Martial Arts in the Development of Chronic Traumatic Encephalopathy” by authors Lim, Ho, and Ho, which was published in the International Journal of Environmental Research and Public Health (2019; 16: 254). I want to congratulate the authors on their case presentation and discussion; chronic traumatic encephalopathy (CTE) is a very important and current issue. I would like to make some contributions to the portion of the authors’ discussion on the topic of “asphyxia” resulting from “neck chokes” in the context of mixed martial arts (MMA) training and competition. In the paragraph of the discussion extending from page 5 to page 6, the authors offer the theory that there is a potential of CTE-related effects, caused by not only head contact and traumatic brain injury but also from “asphyxia.” They present the premise that, over the course of an MMA athlete’s career, repeated momentary episodes of asphyxia from being choked might result in hypoxic ischemic brain injury (HI-BI). They highlight literature establishing that HI-BI occurs in scenarios in which the brain is deprived of oxygen, such as cardiopulmonary arrest, respiratory failure, and carbon monoxide poisoning. Their summation point is that the patient in the presented case exhibited decreased neuropsychological testing performance over time and that HI-BI, resulting from repeated choking, may have contributed to this decline.

An initial issue to address is the terminology used to describe this topic. The authors repeatedly use “asphyxia” to describe the induced physiologic effect from neck compression which occurs as an attempt to submit an opponent during MMA training and competition. Readers could easily misinterpret the situation based on the use of that term. Asphyxia typically describes deprivation of oxygen via obstruction of breathing. Although obstruction of breathing can occur with compression of the airway in the context of MMA as well as grappling training and competition, the goal and typical outcome of neck compression is to occlude the major vasculature, namely, the carotid arteries and jugular veins. This is a more efficient and safer way to make the person submit prior to unconsciousness, which can occur on the order of 5–10 s [1,2]. Although it would most appropriately be referred to medically as a “strangle”, this vascular neck compression is described as a “choke”, and the term has been adopted and accepted by the fighting and grappling communities. The authors use the term choke several times, but repeatedly describe asphyxia, which paints the wrong picture for the reader familiar with medical terminology. One use of the term choke is in their description of the “rear naked neck choke”. In this case, they are referring to the Rear Naked Choke (this compression is described as *Mata Leao* in Portuguese or *Hadaka Jime* in Japanese). They mention that this common and effective choke causes momentary asphyxiation or the opponent to pass out. Although this could rarely be the case, when applied correctly, only the blood flow is cut off, not the airflow. Similarly, because of the interaction between training partners, actual unconsciousness is exceedingly rare.

The other issue to address is the logical gap between the description of the vascular neck compression, or choke, and the presumption of potential neurologic injury. The authors establish that, over the course of an athlete’s career, they will experience many chokes, which is a fair assessment. They describe some of the pertinent compression forces necessary for the jugular veins, the carotid arteries, and the trachea to be collapsed, which are referenced and in line with literature on the topic [1,2,3,4]. However, they next make an unsubstantiated statement: “...HI-BI may develop in the long term in MMA athletes as they are subjected to frequent repeated transient asphyxiation and strangulation...”. The attempt to draw on literature support relies on a loose comparison with several serious prolonged hypoxic scenarios, such as cardiac arrest. The only instance where these comparisons, with extended periods of brain oxygen deprivation, would be appropriate would be a training or competition situation where the competitors and/or referee were acting in a completely nonstandard fashion. The training necessary to learn the choking techniques referred to in this article, both for executing them and for defending against them, is similar across many grappling applications, including but not limited to MMA, jiu jitsu, judo, and catch wrestling. The techniques are typically applied and then stopped when the person being choked “taps” or offers the person applying the technique a verbal or physical signal indicating that the technique has been performed effectively and would render unconsciousness if continued. This interaction between the person executing the choke and the person being choked also extends to live resistance training, where, if a choke is applied effectively in the flow of the struggle, it is subsequently released with a tap prior to unconsciousness. To illustrate the common nature of this practice and the rarity of unconsciousness, I have been choked thousands of times training in similar combat scenarios as the athlete described in this report; I was rendered briefly unconscious only three times, resulting in no subsequent symptoms. My experience is the rule as opposed to the exception. During a formal grappling competition or MMA fight, a choke will be either released when the person being choked taps or submits, or when the referee stops the action due to apparent unconsciousness. Again, if unconsciousness occurs, it is brief and essentially uniformly without persistent symptoms. Given this information, rather than comparing these events to cardiac arrest or complete respiratory failure, a better analogy would be a comparison to presyncope or syncope due to vagal stimulation or orthostasis that improves with brief observation without neurologic sequelae. This would be much less dramatic but much more accurate.

As the authors illustrate, the literature supporting the link between traumatic head injury and CTE is both established and growing. The literature linking CTE or HI-BI and repeat execution of choking techniques in MMA and grappling sports is nonexistent. This lack of evidence is especially significant given the hundreds of thousands of athletes, both past and present, who participate in choke-inclusion sports worldwide. The absence of supporting literature is also significant given the length of time since indexed establishment of the physiologic basis of the presyncope or syncope due to these specific choking techniques, now more than 75 years old [3,5]. I understand and respect the authors bringing up choking techniques in the context of this article, but it would be most reasonable to bring up as a question for possible further study with inclusion of the above information as opposed to likely falsely theorizing that the particular presented patient may have suffered some of his CTE-related neurological decline from repeated chokes.

Thank you to the authors for their manuscript and to the editors for fostering this educational discussion.
